# Clinical strains of *Mycobacterium tuberculosis* exhibit differential lipid metabolism-associated transcriptome changes in *in vitro* cholesterol and infection models

**DOI:** 10.1093/femspd/ftac046

**Published:** 2022-12-12

**Authors:** Kynesha Moopanar, Asanda Nomfundo Graduate Nyide, Sibusiso Senzani, Nontobeko Eunice Mvubu

**Affiliations:** Microbiology, School of Life Sciences, College of Agriculture, Engineering and Science, University of KwaZulu-Natal, Westville Campus, Private Bag X54001, Durban, 4000, South Africa; Microbiology, School of Life Sciences, College of Agriculture, Engineering and Science, University of KwaZulu-Natal, Westville Campus, Private Bag X54001, Durban, 4000, South Africa; Medical Microbiology, School of Laboratory Medicine and Medical Sciences, College of Health Sciences, University of KwaZulu-Natal, 1st floor, Doris Duke Medical Research Institute, Congella, Private Bag 7, Durban, 4013, South Africa; Microbiology, School of Life Sciences, College of Agriculture, Engineering and Science, University of KwaZulu-Natal, Westville Campus, Private Bag X54001, Durban, 4000, South Africa

**Keywords:** *Mycobacterium tuberculosis*, lipid metabolism, clinical strains, cholesterol, transcriptome

## Abstract

Many studies have identified host-derived lipids, characterised by the abundance of cholesterol, as a major source of carbon nutrition for *Mycobacterium tuberculosis* during infection. Members of the *Mycobacterium tuberculosis complex* are biologically different with regards to degree of disease, host range, pathogenicity and transmission. Therefore, the current study aimed at elucidating transcriptome changes during early infection of pulmonary epithelial cells and on an *in vitro* cholesterol-rich minimal media, in *M. tuberculosis* clinical strains F15/LAM4/KZN and Beijing, and the laboratory H37Rv strain. Infection of pulmonary epithelial cells elicited the upregulation of *fadD28* and *hsaC* in both the F15/LAM4/KZN and Beijing strains and the downregulation of several other lipid-associated genes. Growth curve analysis revealed F15/LAM4/KZN and Beijing to be slow growers in 7H9 medium and cholesterol-supplemented media. RNA-seq analysis revealed strain-specific transcriptomic changes, thereby affecting different metabolic processes in an *in vitro* cholesterol model. The differential expression of these genes suggests that the genetically diverse *M. tuberculosis* clinical strains exhibit strain-specific behaviour that may influence their ability to metabolise lipids, specifically cholesterol, which may account for phenotypic differences observed during infection.

## Teaser

Clinical strains of *Mycobacterium tuberculosis* differentially metabolise cholesterol in *in vitro* models.

## Introduction

*Mycobacterium tuberculosis*, the aetiological agent of tuberculosis (TB), which mainly afflicts humans, is at the forefront of global health emergencies (WHO 2021). Certain *M. tuberculosis* bacterial genotypes, such as the Beijing and F15/LAM4/KZN strains, have been most prevalent in XDR and MDR outbreaks (Johnson et al. 2010, Marais et al. 2013) and are examples of members belonging to the *Mycobacterium tuberculosis complex* (MTBC), the current driving force behind the epidemiological success of TB in humans and animals (LoBue et al. 2010, Caminiti et al. 2017).

Apart from being a prominent cell wall component (Vincent et al. 2018, Dulberger et al. 2020), lipids present in the host are a well-documented nutrient source for *M. tuberculosis in vivo*, contributing to its growth and persistence in an otherwise inhospitable environment (Cole et al. 1998, Muñoz‐Elías and McKinney 2006). Alveolar macrophages and pulmonary epithelial cells (PECs) are the primary residence for *M. tuberculosis* bacilli after the inhalation of aerosol droplets, where they remain undetected by the host immune system (Warner and Mizrahi 2007, Barry et al. 2009). Once within the macrophage, *M. tuberculosis* triggers and alters several biochemical pathways to form a lipid-laden milieu, permitting *M. tuberculosis* to thrive in a dormant state (Ehlers and Schaible 2013, Orme and Basaraba 2014). Additionally, the presence of *M. tuberculosis* has been found in fatty tissue surrounding various human organs, implying that the lipids therein are a substantial source of nutrients for *M. tuberculosis* to survive (Neyrolles et al. 2006).

At a genomic level, species and sub-species of the MTBC are closely related; however, they possess varying degrees of abilities in causing the disease (Cole et al. 1998, Coscolla and Gagneux 2010). Considerable advances have been made in elucidating lineage-specific lipid profiles in major *M. tuberculosis* lineages, with mounting evidence that genetic diversity shapes its resultant phenotype (Coscolla and Gagneux 2010). This translates to the need for genetic variability to be accounted for, as it may directly influence the clinical manifestation of TB disease (Gagneux et al. 2006). A multitude of studies have focused on lineage-specific lipid profiles in clinical strains. These include studies that looked at MTBC lineages found to have a higher abundance of lipid-metabolic proteins (Yimer et al. 2020), lipidomic profiling, which revealed an associated increase in transmission and elevated drug resistance (Reed et al. 2007, Ford et al. 2013), and differences in enrichment patterns of host cholesterol pathways (specifically by the Beijing and Unique strains in PECs (Mvubu et al. 2016a,b). Despite this, there still exists a paucity of information concerning this area of *M. tuberculosis* metabolism and thus warrants the need for more comprehensive studies on genotypically diverse drug-resistant strains, especially those dominant in South Africa, a developing country, that features in the top eight countries that are responsible for two-thirds of the world's total cases (WHO 2021).

While studies do exist on *M. tuberculosis* lipid-rich models (Pandey and Sassetti 2008, Griffin et al. 2012, Aguilar-Ayala et al. 2017), there is insufficient work on models mimicking the lipid metabolic state of *M. tuberculosis* clinical strains. Additionally, experimental work on PECs is regrettably sparse, especially because the invasion of *M. tuberculosis* bacilli in these cells has been shown to be important for the establishment of infection (Russell 2001). Therefore, a comprehensive characterisation and analysis of these differentiations is fundamental at a molecular level, particularly in *M. tuberculosis* clinical strains, to account for ‘unique’ lipid profiles that may be responsible for generating phenotypic diversity (Constant et al. 2002, Malaga et al. 2008, Huet et al. 2009, Chiner-Oms et al. 2018).

The purpose of this study was to profile five genes (*choD, fadD28, hsaC, icl1* and *treS*) that were chosen to be evaluated in PEC infection based on their respective roles in lipid metabolism, substantiations of their necessity for *M. tuberculosis* infection and identification as potential virulence genes (Camacho et al. 1999, Murphy et al. 2005, Brzostek et al. 2007, Yam et al. 2009, Shi et al. 2010, Lambrecht et al. 2013), and to additionally elucidate clinically relevant *M. tuberculosis* transcript expression and pathway enrichment profiles in an *in vitro* cholesterol-rich model using RNA-seq technology.

## Materials and Methods

### Ethical clearance

The study was approved by the Biomedical Research Ethics Committee, University of KwaZulu-Natal (reference no. BE078/18).

### *Mycobacterium tuberculosis* strains

Clinically relevant *M. tuberculosis* strains belonging to F15/LAM4/KZN, Beijing and F11 genotype families that were previously isolated from patients in the KwaZulu Natal region (Gandhi et al. 2006, Pillay and Sturm 2007, Chihota et al. 2012) and characterised by the Discipline of Medical Microbiology in University of KwaZulu-Natal were used in the study with permission from Prof. Manormoney Pillay. The genetic identity of the strains was confirmed by IS6110-based Restriction Fragment Length Polymorphism and the drug susceptibility of each strain was determined (Isenberg 1992, Larsen et al. 2007). Two F15/LAM4/KZN strains, KZN605 and KZN1435, were used in the current study, as this genotype was previously associated with high transmission among HIV-coinfected patients in the early-mid 2000s (Pillay and Sturm 2007). The laboratory strain, *M. tuberculosis* H37Rv (ATCC 27294), was used as the virulent control. All *M. tuberculosis*strains were cultured in Middlebrook 7H9 broth, supplemented with 0.5% (vol/vol) glycerol, 0.05% (vol/vol) Tween-80 and 10% (vol/vol) Oleic Acid Albumin Dextrose Catalase (OADC) and incubated at 37ºC in a shaker incubator (1 × g) for 1–2 weeks, unless stated otherwise.

### Seeding and infection of the A549 PECs

The human cell line, pulmonary alveolar epithelial cells (ATCC CCL 185), was employed for use in this study. The cells were revived by addition of 800 μl of passage number 12 stocks to 17 ml of Eagle's Minimum Essential Medium (Lonza, South Africa) and 3 ml of fetal bovine serum (Lonza, South Africa). The cells were maintained to the point of ±90% confluency before being used for the infection assay. Viable cells were counted using a hemocytometer in a trypan blue exclusion test.

PECs were infected in triplicates by each strain of *M. tuberculosis* isolate (H37Rv, KZN605 and Beijing) at a multiplicity of infection (MOI) of 10:1 in 75 cm^2^ tissue culture flasks. Infected cells were subsequently incubated at 37˚C, 5% CO_2_ and 95% humidity. After 4 h post-infection, non-adherent cells were removed and the monolayer was washed three times with Phosphate-buffered saline (PBS) and this was considered as 0 h (Lin et al. 1998). At the 48-h interval, infected cells were lysed with 0.1% Triton X-100. Lysed cells were then centrifuged at 4000 x g for 10 min at 4°C. The pelleted *M. tuberculosis* was washed with PBS and resuspended in 1 ml of TRI Reagent (Zymo Research, South Africa). Thereafter, RNA was extracted from the infecting *M. tuberculosis* strains using the Trizol-phenol–based method coupled with the Direct-zol™ RNA Miniprep Kit (Zymo Research, South Africa), according to the method described below.

### *In vitro* lipid model

An *in vitro* cholesterol model was created using the protocol described by Pandey and Sassetti (2008) and Chang et al. (2009), where clinical strains of *M. tuberculosis* were grown in a minimal medium with the following composition per litre: 0.5 g of asparagine, 1 g of KH_2_PO_4_, 1.5 g of Na_2_HPO_4_, 10 mg of MgSO_4_.7H_2_O, 0.5 mg of CaCl_2_, 0.1 mg of ZnSO_4_, 50 mg of ferric ammonium citrate and 1 ml 1:1 (vol/vol) tyloxapol-ethanol containing 0.01% cholesterol. *Mycobacterium tuberculosis* cells were initially grown in standard Middlebrook 7H9 media and pelleted when the log phase optical density (OD) (0.8–1_600_ _nm_) was achieved. The cells were thereafter washed twice and resuspended with minimal media that was deficient in supplemented cholesterol. This resuspension was used as the inoculum for the cholesterol-rich media, to a growing final volume of 100 ml. A growth curve was elicited by generation of OD_600_ readings over specific time points for both the cholesterol-rich model and *M. tuberculosis* strains grown in standard Middlebrook 7H9 (to be used as a baseline). Colony-forming units per millilitre (CFU/ml) were calculated every 3 days, over a total period of 21 days for both types of media by plating serially diluted cells on Middlebrook 7H11 agar.

### RNA extraction

Total RNA was extracted from *M. tuberculosis* infecting PECs, cholesterol-rich and Middlebrook 7H9 media following a standard Trizol protocol (Thermo Fisher Scientific, South Africa) in conjunction with the Direct-zol™ RNA Miniprep Kit (Zymo Research, South Africa). TRI Reagent (Zymo Research) was added to a bacterial pellet containing Zirconia beads, followed by homogenisation in a Precellys24 Homogeniser (ThermoScientific, South Africa) three times at 1-min intervals. Following lysis, the samples were centrifuged at 15 000 rpm for 1 min at 4°C. The supernatant was transferred into a new tube containing an equal volume of absolute ethanol for the precipitation of RNA, then subjected to a number of washing steps using Direct-zol™ RNA Miniprep Kit buffers and a DNase treatment, before being eluted in DNase/RNase-free water and stored in aliquots at -70°C. RNA concentrations and integrity were assessed with a Nanodrop (ThermoScientific) and 3-(N-morpholino) propanesulfonic acid gel electrophoresis followed by long-term storage at -70ºC, respectively.

Eighteen samples of three biological replicates for each of the *M. tuberculosis* strains, KZN605, Beijing and the laboratory H37Rv grown in 7H9 and lipid-media, were sent for RNA sequencing to the Admera Health institute (New Jersey, USA) based on the respective purities and concentrations. Post-quality control assessment was performed by Admera Health, encompassing RNA Qubit HS Assay (ThermoFisher) for RNA quantification and the Bioanalyzer 2100 Eukaryote Total RNA Nano (Agilent Technologies, CA, USA) for RNA quality evaluation. A sequence library was prepared using the Illumina NEBNext Ultra II with Ribo Zero Plus library preparation kit. The Illumina 2 × 150 HiSeq × 10 platform was used to sequence 60 million paired end reads per sample at 150 bp length.

### Read alignment and transcript assembly

The Illumina-produced raw paired end reads were initially evaluated using FastQC (version 0.11.8; Babraham Bioinformatics, Cambridge, UK). Subsequently, a trimming step was added to filter the raw reads by exclusion of low-quality reads and adapter sequences using Trimmomatic (version 0.36) (Bolger et al. 2014). The resultant trimmed Next Generation Sequencing reads were then mapped to the *M. tuberculosis* H37Rv reference genome using an index constructed by Hierarchical Indexing for Spliced Alignment of Transcripts (version 2.1.0). Each read was mapped with an alignment that fell between 80%–98%, conclusively for the 50 M reads obtained. This produced multiple Sequence Alignment Map (SAM) files, which were converted to the coordinate-sorted Binary Alignment Map file format using the SAMtools software suite (Li et al. 2009). A reference-guided assembler, Stringtie (version 1.2.1), was used to assemble short reads into full-length transcripts (Pertea et al. 2015). Assembled transcripts were combined using the function Stringtie-merge to a resultant unified set of non-redundant transcripts. The input for this function requires a list of Gene Transfer Format files, which are compared with the reference annotation, the *M. tuberculosis* H37Rv genome index. Gffcompare, located within the Stringtie package, employed the use of the merged assembled files and analysed them against the reference to enable discovery and quantification of the novel transcripts. The merged files were then annotated in R Studio (version 1.2.1578) using the Ballgown package to reveal differentially expressed genes.

### Pathway enrichment

The transcripts were subjected to further analysis in the Ballgown package, to determine gene fold changes, q and *P* values between the *M. tuberculosis* clinical strains grown in cholesterol media versus the *M. tuberculosis* clinical strains grown in Middlebrook 7H9 broth. The results were filtered using a fold change cut-off value of ≥2 (to indicate a 2-fold upregulation) and ≤0.5 (to indicate a 2-fold downregulation) to identify significant genes for biological pathway enrichment analysis using the BioCyc and Kyoto Encyclopedia of Genes and Genomes metabolic databases.

### Quantitative reverse transcription PCR

The expression of the lipid-associated virulence genes that were chosen to be profiled, during *M. tuberculosis* infecting PECs, was determined. A total of three clinical strains (H37Rv, KZN605 and Beijing) were evaluated in this case. Gene sequences, in FASTA format, were retrieved from the Mycobrowser database (Kapopoulou et al. 2011) and thereafter used as the template sequence in the Primer3Plus program to generate the best primer pair adequate for quantitative reverse transcription (qRT)-PCR.

The cDNA was synthesised from the extracted RNA using iScript cDNA synthesis kit (Bio-Rad, South Africa) according to the manufacturer's instructions. For standardisation, a total of 1 μg of RNA sample was used for the reaction. The cDNA samples were then stored at -20°C until further use.

The expression of lipid-associated metabolic genes was quantified using Ssoadvanced Universal SYBR Green Supermix kit (Bio-Rad Laboratories, Hercules, CA, USA) with the reaction being carried out in a CFX96 Real-Time System (Bio-Rad). The cycling conditions were as follows: a holding stage at 95˚C for 3 min; and a PCR stage of 40 cycles, which included 95°C for 30 s, 60°C for 30 s and 75°C for 30 s. The melt curve analysis was determined at continuous fluorescence set at 90°C for 1 min, 60°C for 30 s and 95°C for 15 s. Each 10 µl reaction was performed according to the manufacturer's instructions (Bio-Rad) in three biological replicates. Determination of relative gene expression was normalised to the 16S rRNA housekeeping gene. Fold changes were calculated using the 2^–∆∆Ct^ method and these data generated the heat map using MeV software (Saeed et al. 2003).

### Statistical analysis

Significant differences in the expression of lipid-associated genes during RT-qPCR among clinical strains of *M. tuberculosis* relative to the laboratory H37Rv strain were analysed by Students *t*-test, in conjunction with ANOVA, in GraphPad Prism 7. A *P* value of less than 0.05 was considered statistically significant (Yuan et al. 2006).

## Results

### *In vitro* lipid-associated transcript expression is variable among genetically diverse clinical strains of *M. tuberculosis*

**Figure 1.**
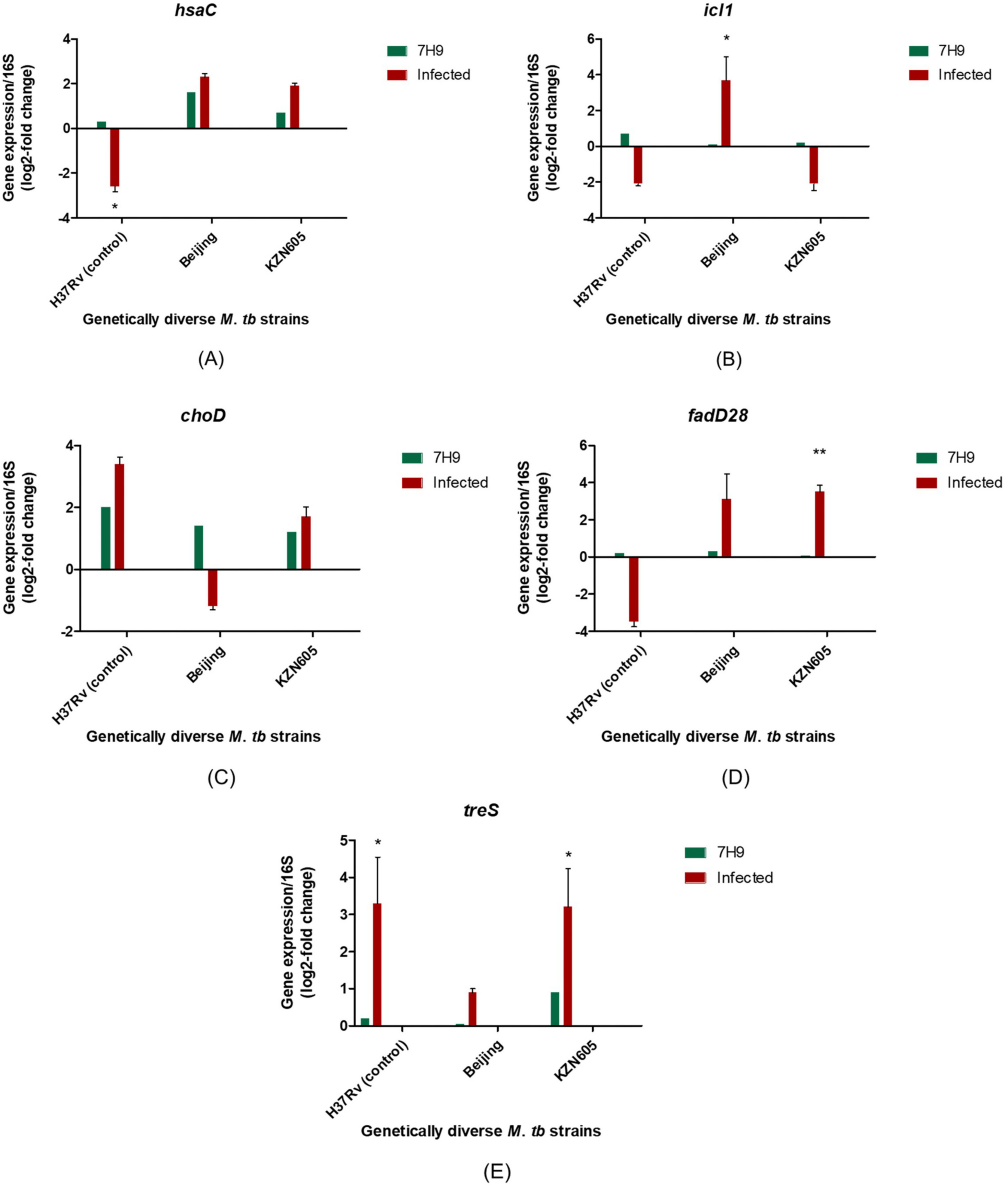
Differential expression of *Mycobacterium tuberculosis*(*M. tuberculosis*) lipid-associated virulence genes in Middlebrook 7H9 culture medium and during 48-h pulmonary epithelial cell infection. Selected genes have been identified as being vital in lipid metabolism in *M. tb* during infection. (**A-E**) qRT-PCR bar graphs depicting the gene expression values as a log2-fold change for the various lipid-associated virulence genes in various clinical strains of *M. tb* in standard Middlebrook 7H9 broth and during 48-h infection of PECs. Statistical significance is represented by one asterisk (*P* ≤ 0.05) and two asterisks (*P* ≤ 0.01) as per a non-parametric Mann-Whitney *t*-test. Relative gene expression was quantified using the 2^−ΔΔCt^ method.

Previously, we observed high enrichment of cholesterol biosynthesis pathways during early infection of PECs by Beijing strains (Mvubu et al.2016a), hence transcripts (*hsaC, icl1, choD, fadD28, treS*) implicated in fatty acid and cholesterol metabolism were selected for quantification through RT-qPCR for clinical strains of *M. tuberculosis*. Variation in gene expression was observed among clinical strains for the *hsaC* (Fig. 1A) transcript compared with the H37Rv strain. The Beijing and KZN605 strains exhibited high expression when compared with the laboratory strain, *M. tuberculosis* H37Rv, during infection of PECs. When assessing both conditions, *hsaC* transcript expression was significantly (*P* = 0.0361) downregulated in H37Rv-uninfected cells compared with 7H9 broth. Furthermore, no significant differences (*P* > 0.05) were observed in *hsaC* expression for both Beijing and KZN605 between 7H9 and during infection of PECs. The *icl1* fold changes were exceedingly high for the Beijing strain (*P* = 0.0418) compared with the H37Rv and KZN605 strains (Fig. 1B) in the infection model. H37Rv, KZN605 and Beijing exhibited very low expression in 7H9 medium. Gene expression values for the *choD* gene in 7H9 medium (Fig. 1C) indicate higher fold changes induced for H37Rv during infection than in 7H9 broth. Although not significant, *choD* showed low level expression during infection for Beijing and KZN605 when compared with H37Rv. The Beijing and KZN605 strains exhibited comparable fold changes for the *fadD28* transcript (Fig. 1D) when compared with the H37Rv control during normal culturing conditions. The Beijing and KZN605 (*P* = 0.0062) strains, however, induced higher expression of *fadD28* when compared with the H37Rv strain during infection. Overall, *fadD28* was greatly expressed during infection when contrasted with the expression values from the 7H9 medium. The fold changes in the *treS* gene (Fig. 1E) were much lower for the Beijing strain when compared with KZN605 (*P* = 0.0219) and the H37Rv control (*P* = 0.0488) during 48-h infection. Overall, expression for this gene was highly induced in infection as opposed to standard culturing conditions.

The fold change for qRT-PCR analysis was calculated using the Ct values of the lipid genes from the clinical strains infecting PECs versus Ct values of lipid genes in clinical strains cultured in 7H9 broth. Statistical significance is represented by one asterisk (*P* ≤ 0.05) and two asterisks (*P* ≤ 0.01) as per a non-parametric Mann-Whitney *t*-test. Error bars represent the ±SD for three biological replicates for each strain. The 16S rRNA gene was used as an internal standard to normalise gene expression data.

**Figure 2.**
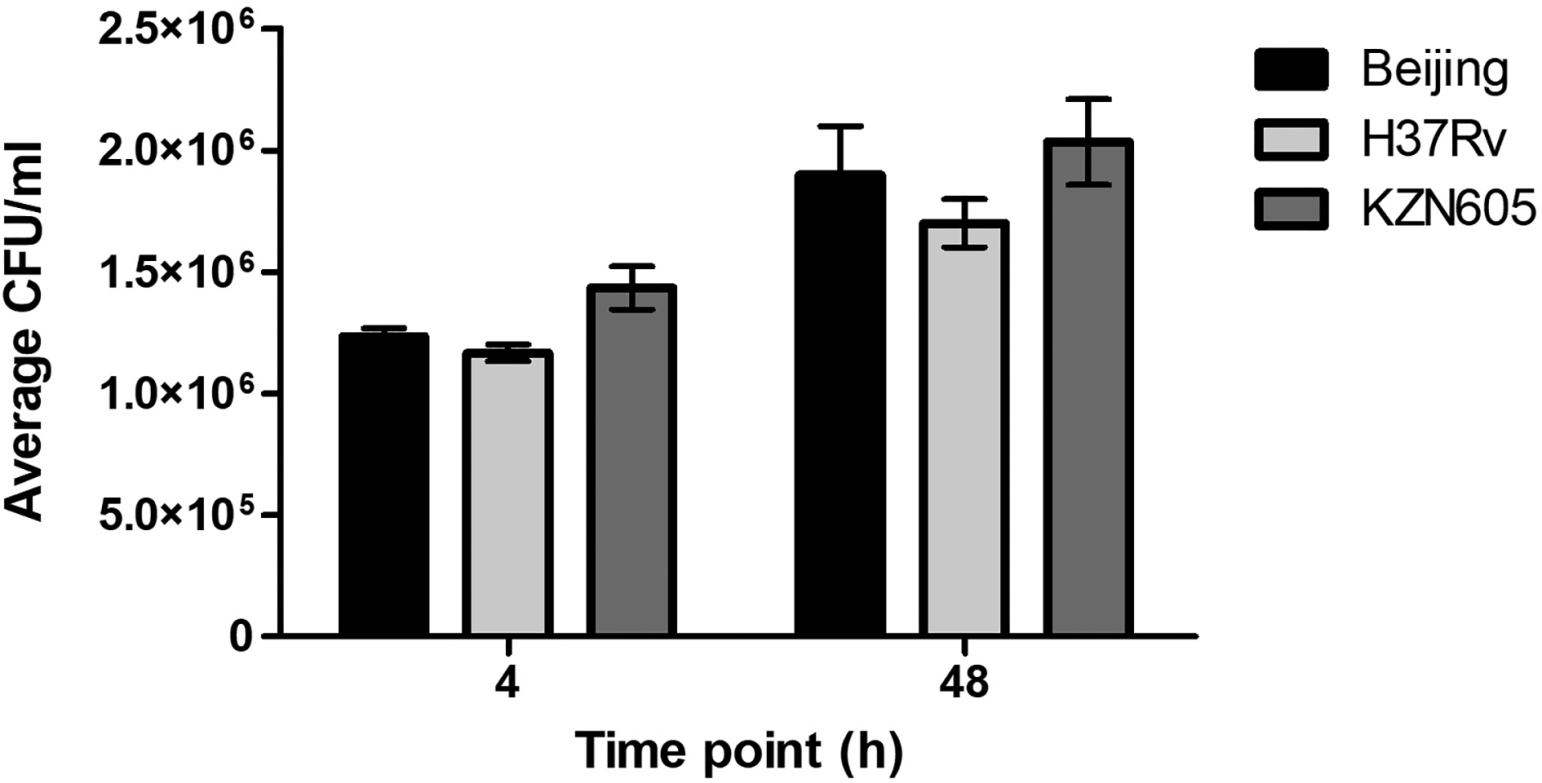
Graphical representation of the average biological assay 1, 2, and 3 bacterial colonies (CFU/ml) from clinical *M. tuberculosis* strains, Beijing and F15/LAM4/KZN, and the laboratory strain H37Rv, post-infection in pulmonary epithelial cells at 4 and 48 h, respectively.

To assess whether changes in gene expression were due to differences in intracellular growth of clinical strains of *M. tuberculosis*, PECs were lysed and intracellular bacterial cells were enumerated in 7H11 Middlebrook agar plates. The clinical strains exhibited differential intracellular growth in alveolar macrophages (Fig. 2), with the highest by the F15/LAM4/KZN strain at both 4- and 48-h post-infection. However, these differences were not significant (*P* > 0.05), hence *M. tuberculosis* gene expression may be due to strain-specific virulence traits, rather than their growth patterns in PECs.

### Growth rates of *M. tuberculosis* clinical strains cultivated in an exclusively cholesterol environment and in standard liquid growth medium reveal KZN605 and Beijing to be slow growers

**Figure 3.**
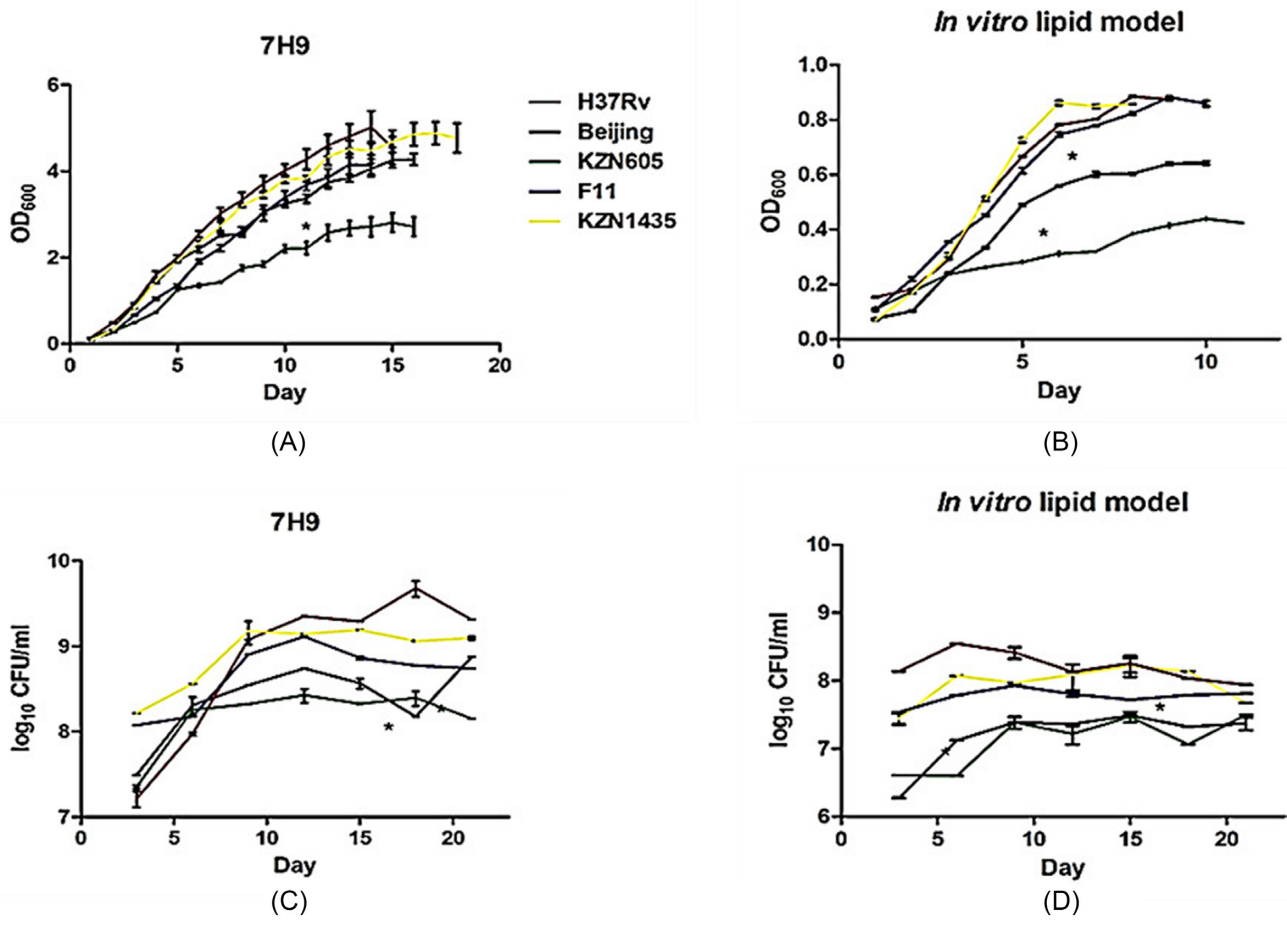
Growth curves and bacterial CFU/ml of clinical *M. tuberculosis* strains. (**A**) Growth curve for *M. tuberculosis* strains cultivated in 7H9; (**B**) growth curve for *M. tuberculosis* strains cultivated in the *in vitro* lipid model; (**C**and**D**) the corresponding CFU/ml for each type of environment. For the growth curve, growth was plotted on a log_10_ scale by measurement of OD_600nm_ and recorded every 2 days until a stationary phase was reached. The bacterial CFU/ml counts were determined by plating serially diluted *M. tuberculosis* on Middlebrook 7H11 agar for a total of 21 days. The curves illustrated correspond to the mean ± SD of three biological replicates. The *M. tuberculosis* H37Rv laboratory strain and the *M. tuberculosis* KZN1435 clinical strains grew optimally in both culturing conditions, while the *M. tuberculosis* KZN605 and Beijing strains presented the slowest growth. * denotes differences that are statistically significant (*P* < 0.05) as per a non-parametric Mann-Whitney *t*-test.

Fast-growing mycobacteria have been well established as utilising sterols, such as cholesterol, to multiply and synthesise energy for the bacterium (Martin 1977, Mahato and Garai 1997, Brzostek et al. 2007). The growth rates of the *M. tuberculosis* strains grown in media supplemented with 0.01% cholesterol compared with the *M. tuberculosis* strains grown in standard Middlebrook 7H9 medium are depicted below (Fig. 3A and B). The findings indicate that the H37Rv and KZN1435 strains grew optimally in 7H9 and in lipid media, while the Beijing and KZN605 strains lacked in growth compared with other strains. The F11 and Beijing strains grew similarly to the H37Rv and KZN1435 strains in 7H9; however, in lipid media, only the F11 strain was comparable with H37Rv and KZN1435 in growth patterns, while Beijing grew to a lower density than all three. These results were confirmed by determining the number of viable cells every third day for 21 days and generation of the CFU/ml graphs (Fig. 3C and D). The number of viable cells for each clinical strain in both types of media mirror the growth patterns illustrated in Fig. 3A and B. Additionally, it is also confirmed that the *M. tuberculosis* clinical strains do not exhibit any special growth characteristics in cholesterol media, as indicated by the comparable growth patterns relative to the 7H9 medium. As anticipated, the maximal cell density for *M. tuberculosis* grown in a defined medium with cholesterol as its primary carbon source was lower than that of *M. tuberculosis* grown in an OADC-rich 7H9 medium. This could also be attributed to the restrictive low concentration of cholesterol added to the medium, due to cholesterol being difficult to solubilise. The extended lag phase discerned for the clinical strains in cholesterol media is likely due to the strains taking time to acclimatise to a new metabolic state. Furthermore, low OD detected in cholesterol-rich minimal media might be due to the formation of small sized mycobacterial cells, as reported in nutrient-starved mycobacterial cells, that is caused by *M. tuberculosis* morphological plasticity (Berney and Cook 2010, Shleeva et al. 2011, Wu and Dick 2015, Wu et al. 2016).

### The transcriptional profile of differentially expressed genes in clinical strains of *M. tuberculosis* under cholesterol conditions and delineation of accompanying pathways

**Figure 4.**
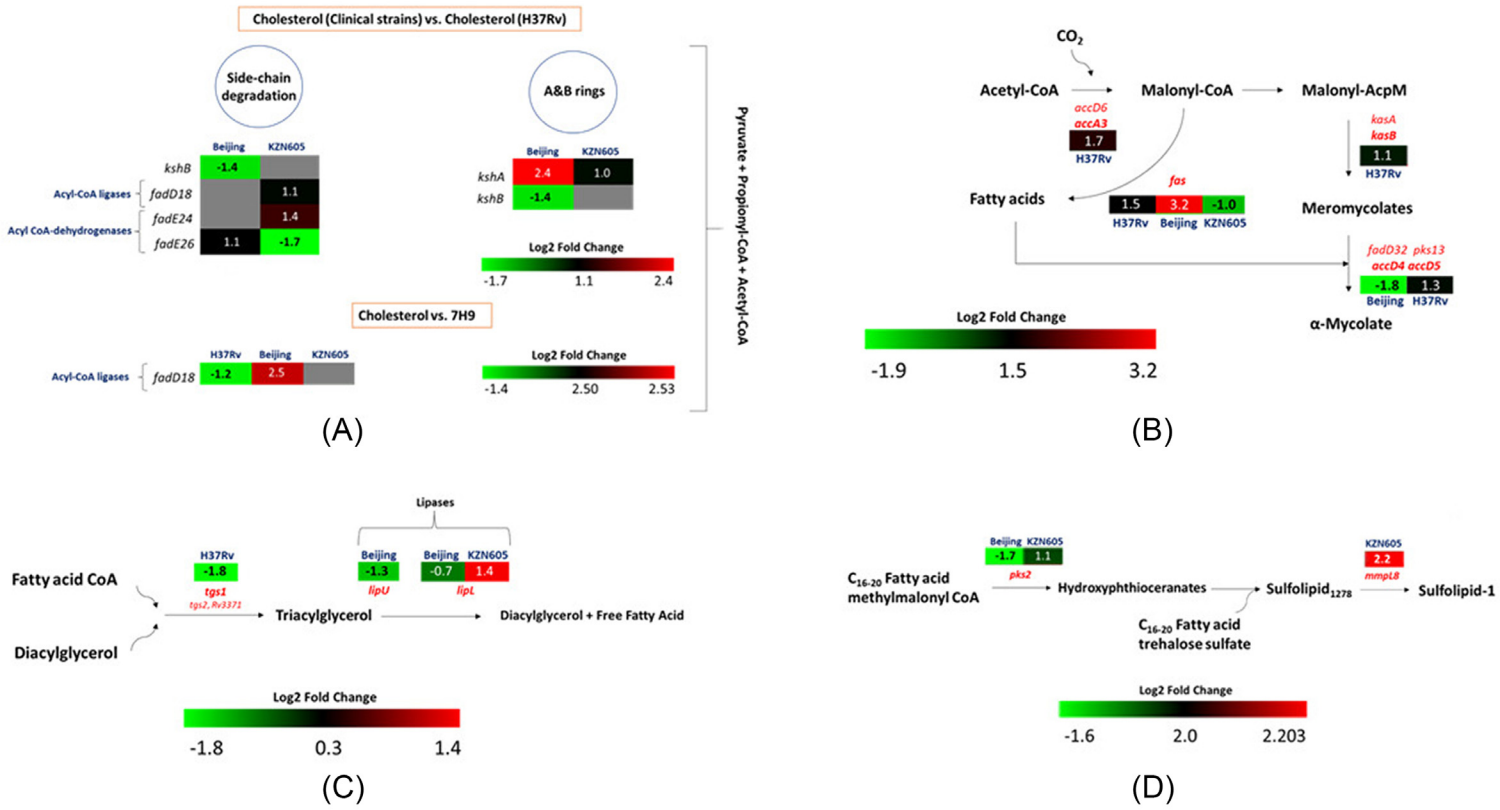
(**A**) Compilation of functionally categorised genes involved in the breakdown of cholesterol and its relative expression. The transcriptomic data of *M. tuberculosis* clinical strains KZN605 and Beijing were compared with the reference H37Rv laboratory strain in an *in vitro* lipid model and while additionally comparing conditions of *M. tuberculosis* strains (H37Rv, Beijing, KZN605) grown in cholesterol-supplemented minimal media against the same strains grown in 7H9/10% OADC medium. Genes were grouped together according to the varying functions each plays in the multiple stages of cholesterol degradation. (**B**) Characterisation of a fraction of the mycolic acid synthesis pathway in *M. tuberculosis* strains. Gene expression implicated in the mycolic acid synthesis pathway in *M. tuberculosis* strains KZN605, Beijing and H37Rv in an *in vitro* lipid model compared with the KZN605, Beijing and H37Rv strains in Middlebrook 7H9 medium. (**C**) The simultaneous depiction of triacylglycerol synthesis and degradation in *M. tuberculosis* strains and the concomitant differential expression of genes involved in these processes. Gene expression implicated in the triacylglycerol synthesis and degradation pathway in *M. tuberculosis* strains KZN605, Beijing and H37Rv in an *in vitro* lipid model compared with the KZN605, Beijing and H37Rv strains in Middlebrook 7H9 medium. (**D**) Gene expression implicated in the sulfolipid-1 synthesis pathway in *M. tuberculosis*. The differential expression of genes involved in the production of sulfolipids in *M. tuberculosis* strains H37Rv, Beijing and KZN605 in an *in vitro* lipid model compared with the same strains in 7H9 medium. The results are presented as log2-fold change in expression in a heatmap format. A colour key is provided to indicate log2-fold changes, and grey boxes indicate genes that were not detected from the transcriptome data.

To investigate the up- or downregulation of lipid metabolic genes in diverse clinical strains of *M. tuberculosis*, a transcriptional profile was constructed to enable a comparative analysis of gene expression levels in *M. tuberculosis* grown in enriched minimal media with cholesterol as an additive versus 7H9 medium. R Studio and the Ballgown package provided the fold change data. If the fold changes fell within the range of ≥1 and ≤0.5, they were deemed as up- and downregulated, respectively. An additional criterion of a *P* ≤ 0.05 was added, but was not strictly adhered to (Tables 1 and 2). To accurately assess and identify genes implicated in lipid metabolism, Mycobrowser (Kapopoulou et al. 2011) was used to functionally categorise genes. These results prompted the further classification of lipid metabolic-associated genes into specific lipid-metabolic pathways, particularly those that are involved in cholesterol degradation, using BioCyc databases. Using the aforementioned method, biological pathways were generated as seen below (Fig. 4).

**Table 1.** Genes that are up- and downregulated in the three *M. tuberculosis* strains grown in cholesterol media relative to those grown in 7H9.

	H37Rv		Beijing		KZN605	
Gene	FC	*P*	FC	*P*	FC	*P*
*fadD18*	0.4251	0.6795	5.7565	0.0560	–	–
*accA3*	3.3295	0.0037*	–	–	–	–
*kasB*	2.1862	0.8273	–	–	–	–
*fas*	2.7633	0.5251	8.9266	0.3546	0.4999	0.0833
*accD4*	–	–	0.2855	0.5152	–	–
*accD5*	2.4895	0.5463	–	–	–	–
*tgs1*	0.2885	0.6359	–	–	–	–
*lipU*	–	–	0.4034	0.7708	–	–
*lipL*	–	–	0.6253	0.2718	2.5924	0.0129*
*mmpL8*	0.2918	0.0286*	–	–	–	–

FC: fold change; * denotes differences that are statistically significant (*P* < 0.05); a dash (–) indicates that the transcript fell below the threshold for detection.

**Table 2.** Genes that are up- and downregulated in the two *M. tuberculosis* clinical strains relative to the H37Rv strain in cholesterol media.

	Beijing		KZN605	
Gene	FC	*P*	FC	*P*
*kshA*	5.3141	0.0132*	2.061	0.9737
*kshB*	0.3753	0.0349*	–	–
*fadD18*	–	–	2.0817	0.0947
*fadE24*	–	–	2.7014	0.0031*
*fadE26*	2.2014	0.2603	0.3007	0.3279
*pks2*	0.3111	0.0151*	2.1721	0.4251
*mmpL8*	–	–	4.6050	0.2682

FC: fold change; * denotes differences that are statistically significant (*P* < 0.05); a dash (–) indicates that the transcript fell below the threshold for detection.

The genes assessed in the PEC infection model (*choD, hsaC, icl1, fadD28* and *treS*) were not detected in the RNA-seq data, leading to the conclusion that these genes were expressed at an exceptionally low level to even be observed, and are not activated when subjected to a sole cholesterol environment. Genes that were directly involved in the degradation of cholesterol were then evaluated. In addition to relating the cholesterol degradation data to transcriptional changes of *M. tuberculosis* occurring within a standard 7H9 medium, the data of the *M. tuberculosis* clinical strains grown in a cholesterol-induced environment were related to the transcriptome of *M. tuberculosis* H37Rv, also grown in cholesterol (Fig. 4A), to distinguish any differences between the wild-type and clinical strains. Over 200 genes in *M. tuberculosis* have been reported to be regulated by cholesterol, these genes belonging to various regulons such as the KstR1 regulon (Kendall et al. 2010), located within the Cho region and the SigE regulon (Fontán et al. 2008). In this study, two genes (*kshB* and *fadE26*) of the KstR1 regulon and one gene (*fadE24*) of the SigE regulon were induced in either or both clinical strains (Beijing and KZN605) in the data analysis of *M. tuberculosis* clinical strains grown in cholesterol with the *M. tuberculosis* H37Rv wild-type strain also grown in cholesterol as the reference (Fig. 4A). The data for the experiment of *M. tuberculosis* strains subjected to cholesterol versus *M. tuberculosis* strains grown in 7H9/10% OADC medium induced changes on one gene belonging to the KstR1 regulon (*fadD18*) (Fig. 4A). Cholesterol degradation engages with three integral pathways, which are: (a) β-oxidation of the cholesterol side chain; (b) cleavage of the A and B rings; and (c) degradation of the C and D rings, of which the former two are highlighted in the results of this study (Wipperman et al. 2014). The first step of the alkyl side-chain degradation is a necessary one to initiate the process and is overseen by the cytochrome P450 genes and *kshB*, with *kshB* simultaneously catalysing the breakdown of the A and B rings (Cole et al. 1998, Johnston et al. 2010). Compared with the *M. tuberculosis* H37Rv strain grown in cholesterol, *kshB* was significantly downregulated for the Beijing strain. Conversely, the *kshA* gene, also implicated as a co-enzyme in A and B ring degradation (Capyk et al. 2011), exhibited upregulation for both the Beijing (significant) and KZN605 strains (Fig. 3A). *Mycobacterium tuberculosis* possesses 36 acyl-CoA ligases (Nesbitt et al. 2010). In the present study, the expression data for *M. tuberculosis* clinical strains compared with *M. tuberculosis* H37Rv in cholesterol media (Fig. 4A) shows that one gene was induced. In the experimental data, with 7H9 medium as the reference, the expression of one acyl-CoA ligase was induced. The *fadD18* gene was shown to be downregulated in H37Rv and upregulated in Beijing (Fig. 3A). To complete the first cycle of β-oxidation, β-dehydrogenation takes place on a fatty acyl-CoA thioester, which is catalysed by an acyl-CoA dehydrogenase (Wipperman et al. 2014, Yang et al. 2015). Results demonstrate that the clinical strains of *M. tuberculosis* relative to H37Rv in cholesterol media induced the expression of two acyl-CoA dehydrogenase genes. For the Beijing strain, *fadE26* was upregulated. In KZN605, *fadE26* was downregulated, while *fadE24* (significantly) was upregulated (Fig. 4A).

Through RNA-seq analysis, it was possible to define other metabolic pathways related to the channelling of the metabolites produced from cholesterol degradation. Transcriptional responses of genes involved in mycolic acid synthesis were determined in the *M. tuberculosis* strains cultivated in cholesterol relative to the strains grown in 7H9 medium (Fig. 4B). One such factor present in the pool of resulting metabolites from the breakdown of cholesterol is acetyl-CoA (Yang et al. 2009). The *accA3* gene, one of three acyl-CoA carboxylases in mycobacteria, is primarily responsible for the conversion of acetyl-CoA to malonyl-CoA, to be used in either the FAS I or FAS II system as an elongation unit (Oh et al. 2006). This gene was shown to be significantly upregulated in H37Rv. The malonyl-CoA unit, upon entering the FAS I system, relies singularly on the catalytic activities of the *fas* gene to transform it into a free fatty acid that subsequently partakes in mycolic acid synthesis (Zimhony et al. 2004). According to the results, the *fas* gene was upregulated in H37Rv and Beijing but downregulated in KZN605. In the FAS II process, the elongation of the acyl-CoA to be incorporated into the meromycolate chain is performed by several enzymes, one such enzyme being KasB (Slayden and Barry 3rd 2002). The *kasB* gene, encoding for this enzyme, was found to be downregulated in the Beijing strain when compared with the laboratory H37Rv strain. The carboxylation of the merochain is catalysed by *accD4*, with *accD5* forming part of the complex (Portevin et al. 2005). The *accD4* gene was downregulated in Beijing, while, by contrast, *accD5* was upregulated in H37Rv. Seemingly, the promotion of mycolic acid synthesis is higher in the wild-type strain as opposed to the clinical strains.

The synthesis of triacylglycerol (TAG) is an indicator for *M. tuberculosis* entering a dormancy state, as TAG is utilised under these conditions as a reservoir for energy (Daniel et al. 2004, Deb et al. 2009). The *tgs1* gene, which is functional in this process, was shown to be downregulated in H37Rv, meaning that TAG synthesis was not initiated for this strain (Fig. 4C). The Beijing strain exhibited similar results, in that the dissemination of TAG was not endorsed, as seen by the downregulation of the *lipU* and *lipL* genes. However, the *lipL* gene showed significant upregulation for the KZN605 strain.

In terms of the synthesis of the cell wall sulfolipid, the *pks2* gene was shown to be downregulated for the Beijing (significantly) and upregulated in KZN605 strains when compared with these strains in 7H9 medium (Fig. 4D). The essential sulfolipid transporter gene, *mmpl8*, was upregulated for the KZN605 strain.

### qRT-PCR validation of RNA-sequencing selected genes in both growth conditions

**Figure 5.**
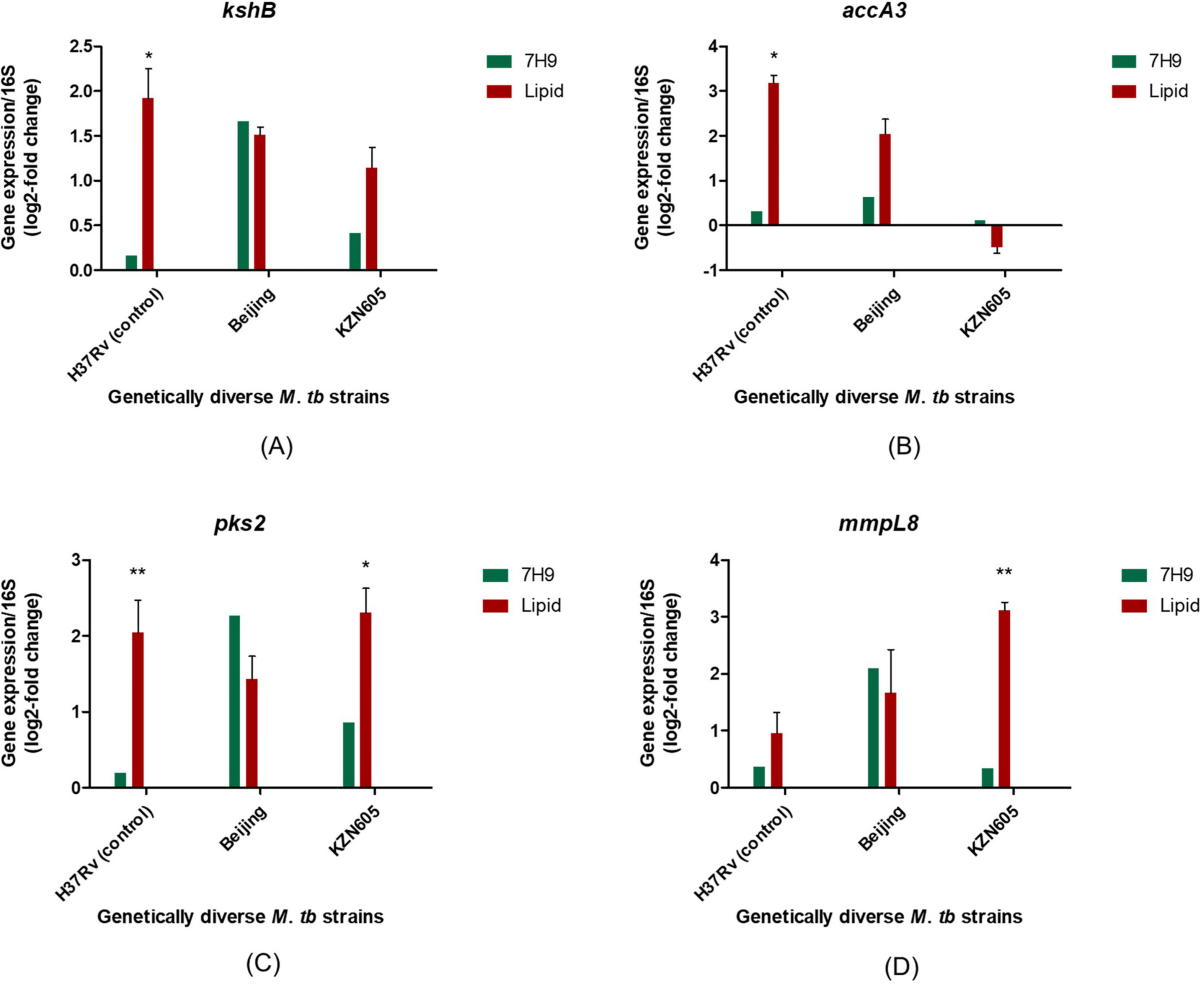
Differential expression of selected *M. tuberculosis* (*M. tb*) lipid-associated virulence genes in Middlebrook 7H9 culture medium and cholesterol-supplemented media for functional confirmation of RNA-sequencing analysis. (**A-D**) qRT-PCR bar graphs depicting the gene expression values as a log2-fold change for the various lipid-associated virulence genes in various clinical strains of *M. tuberculosis* in standard Middlebrook 7H9 broth and lipid media. Relative gene expression was quantified using the 2^−ΔΔCt^ method. The fold change for qRT-PCR analysis was calculated using the Ct values of the lipid genes grown in 0.01% cholesterol versus Ct values of lipid genes in clinical strains cultured in 7H9 broth. Statistical significance is represented by one asterisk (*P* ≤ 0.05) and two asterisks (*P* ≤ 0.01) as per a non-parametric Mann-Whitney *t*-test. Error bars represent the ± SD for three biological replicates for each strain. The 16S rRNA gene was used as an internal standard to normalise gene expression data.

To validate the transcriptomic data obtained from RNA-sequencing, four genes of interest were selected for quantification of gene expression in *M. tuberculosis* clinical strains using a genotypic assay, qRT-PCR. The *kshB* gene showed a higher level of gene expression in H37Rv (*P* = 0.0458) and KZN605, but lower in Beijing relative to its 7H9 counterparts (Fig. 5A). The *accA3* gene was induced highly in H37Rv (*P* = 0.0226) and Beijing and exhibited low expression in KZN605 (Fig. 5B). The *pks2* gene exhibited higher gene expression in H37Rv (*P* = 0.0093) and KZN605 (*P* = 0.0184), while having a decreased expression in the Beijing strain (Fig. 5C). The expression of the *mmpL8* gene was induced highly in all strains and significantly in KZN605 (*P* = 0.0073), except for the Beijing strain, where it was lowly expressed (Fig. 5D). Overall, a complementation of data was demonstrated between RNA-sequencing analysis and qRT-PCR, for all the selected genes.

## Discussion

There is increasing evidence that genetically diverse clinical strains of *M. tuberculosis* exhibit strain-specific phenotypes in both *in vitro* and *in vivo* models (Manca et al. 1999, 2001, Shanley et al. 2018). This diversity may be related to specific genotypic and phenotypic characteristics that are associated with virulence in *M. tuberculosis* (Kato-Maeda et al. 2001, Malik and Godfrey-Faussett 2005, Nicol and Wilkinson 2008). Studying genetically diverse clinical strains permits accessibility to evaluating genes that are potentially differentially expressed and presents an opportunity to link this genetic variation to the effects on the level of transcripts expressed (Gao et al. 2005). Lipid molecules are a well-documented source of nutrient that *M. tuberculosis* harnesses to augment growth and as such is considered an important virulence factor (Lovewell et al. 2016, Aguilar-Ayala et al. 2017). The lipid nature of the cell wall and its differential expression is also a determinant of whether *M. tuberculosis* infection will enter a chronic phase of infection. Certain lipids in *M. tuberculosis* are associated with a corresponding immunological response and due to its inherent plasticity, *M. tuberculosis* will activate it accordingly (Queiroz and Riley 2017). A vast network of mycobacterial virulence genes provides *M. tuberculosis* with the tools to synthesise and degrade various lipids, dedicating no less than 250 genes (Cole et al. 1998). Due to the complexity of this lipid breakdown process and phenotypic variability present in clinical strains of *M. tuberculosis*, it is essential to study these transcriptomic changes in a controlled and defined condition as opposed to using standard conditions *in vitro*. To achieve this, the current study aimed to profile clinical mycobacterial responses to a cholesterol-rich (one of the most abundant lipids found in the host cell (Wayne and Hayes 1996)) environment as well as during PEC infection and how this might potentially affect the distribution and dominance of these genotypes worldwide (Cox et al. 1999, Rindi et al. 2002, Muñoz-Elías and McKinney 2005, Brzostek et al. 2007, Griffin et al. 2011).

### Genetic diversity of clinical strains may confer differences in expression of lipid-associated virulence genes in *M. tuberculosis*

The *hsaC* gene encodes the HsaC enzyme, which is actively involved in cholesterol degradation (Van der Geize et al. 2007). This gene was upregulated when *Mycobacterium bovis*was exposed to a cholesterol-rich environment in a liquid medium. The same study also demonstrated expression of this gene in *Escherichia coli*. The HsaC enzyme preferentially metabolised cholesterol over biphenyl metabolites and pyruvate (Van der Geize et al. 2007). In the current study, the higher fold changes of *hsaC* for Beijing and KZN605 in 7H9 medium and similarly post 48-h infection of PECs is noted (Fig. 1A). It has been well established that the sequestering of cholesterol during pathogenesis transpires during the chronic phase of infection (Pandey and Sassetti 2008) and during macrophage infection (Schnappinger et al. 2003). Furthermore, it was proven in animal models of infection that when the *hsaC* gene in *M. tuberculosis* H37Rv was knocked out, the resulting mutant strain caused a slower dissemination of bacilli, decreased lesions in the granuloma and had persisted to a lesser extent than that of the wild-type strain (Yam et al. 2009). The increased expression of this gene in clinical strains of *M. tuberculosis* translates to the active degeneration of cholesterol during early infection (where cholesterol is a key nutrient at this stage (Chang et al. 2009, Yam et al. 2009)) in PECs and can be considered an important virulence factor. Furthermore, lipid raft structures have been described to be aggregated by *M. tuberculosis* in the PEC plasma membrane, which is rich in cholesterol and other compounds, making this potentially an accessible source of host cholesterol for degradation (Fine-Coulson et al. 2012).

The *icl1* gene in *M. tuberculosis* encodes for isocitrate lyase 1 and partakes in the glyoxylate shunt, which aids in the utilisation of even-chain fatty acids, and in a double capacity also catalyses the last step of the methylcitrate cycle (McKinney et al. 2000, Muñoz-Elías and McKinney 2005). Remarkably, a high fold change for the *icl1* gene was observed in the Beijing strain during infection alone (Fig. 1B). This gene has prominently been implicated and upregulated in the lungs of mice during *M. tuberculosis* infection when adaptive immunity is triggered (Timm et al. 2003, Shi et al. 2010) and highly expressed in *in vitro* culture experiments, where *M. tuberculosis* had been isolated from the sputum of patients (Garton et al. 2008). Additionally, *icl1* in the H37Rv wild-type strain was found to be upregulated in a cholesterol as a sole carbon source environment (Pawełczyk et al. 2021). It is postulated that during this infection process, the low fold changes for *icl1* in KZN605 (Fig. 1B) may be due to propionyl-CoA (derived from cholesterol degradation) being channelled into the methylmalonyl-CoA pathway, rather than the methylcitrate cycle (Muñoz-Elías and McKinney 2005).

Despite being a non-essential gene in direct cholesterol breakdown, there is abundant evidence that *choD* is unambiguously required for the intracellular survival of *M. tuberculosis* and is capable of modulating host immune responses (Brzostek et al. 2007, Klink et al. 2013). To this end, the evaluation of *choD* expression in A549 PECs proved to be worthy of investigation. The results indicate that the expression of *choD* was markedly lower in 7H9 medium than during infection for the H37Rv control (Fig. 1C). This is in line with the research that *M. tuberculosis choD* shows higher expression in the lungs of mouse than broth culture (Dubnau et al. 2005). Moreover, *choD* exhibited low expression for the KZN605 and Beijing strains during PEC infection, in stark contrast to the laboratory control, the H37Rv strain (Fig. 1C). The importance of *choD* expression in lungs for the *M. tuberculosis* H37Ra wild-type was demonstrated by Brzostek et al. (2007), where a CFU count of viable bacteria post 10 weeks of infection of a mutant *choD* strain showed a complete clearing of *M. tuberculosis* from the lungs of mice. This corroborates with the H37Rv *choD* expression data presented in this experimental work. Interestingly, *choD* exhibited a downregulation in the Beijing strain, post 48-h infection, despite being shown to be a key enzyme that drives pathogenesis in mice infection experiments, such as infection of peritoneal macrophages (Brzostek et al. 2007). Our study shows that for the infection of PECs at the very least, the Beijing strain does not require the activation of this gene during early infection, which might be a strain-specific trait in this cell line. Because the expression of *choD* has been shown to diminish Toll-like receptor 2 (TLR2) signalling (Klink et al. 2013), the lower expression of *choD* in clinical strains proves advantageous in that TLR2 signalling is not influenced in alveolar epithelial cells, and innate immunity is unhindered by the expression of this gene during infection. Nevertheless, for a hypervirulent Beijing strain, it has been demonstrated that its lipid fractions induced the downregulation of TLR2, which means that this immune response is affected for this strain in any case (Rocha-Ramírez et al. 2008).

The *fadD28* gene, one of the FadD paralogues, is involved in the production of phthiocerol dimycocerosate (PDIM). This lipid has been found to be connected to the virulence of mycobacteria (Camacho et al. 1999, Pethe et al. 2004, Stewart et al. 2005) and stunting of phagosome maturation (Astarie-Dequeker et al. 2009). The lower fold changes noted for the strains grown in broth culture were anticipated (Fig. 1D), as stated by Domenech and Reed (2009), where they observed the possibility of *M. tuberculosis* to lose its innate ability to synthesise PDIM, especially if the stocks used have been *in vitro* cultivated for a prolonged time. Conversely, the *M. tuberculosis* strains, KZN605 and Beijing, showed higher fold changes for *fadD28* than H37Rv (Fig. 1D). Several mutagenesis studies (Camacho et al. 1999, Cox et al. 1999) have shown substantial evidence that PDIM-mutants (*fadD28* included) prompted attenuation in mouse models of infection, leading to the conclusion that this gene is important for the infection process, which is in agreement with the results observed.

The disaccharide, trehalose, plays a major role in the transportation of mycolic acids. The enzyme TreS, as encoded for by the *treS* gene, catalyses the interconversion of maltose and trehalose (De Smet et al. 2000), and is shown to be required for late-stage pathogenesis in mice models of infection (Murphy et al. 2005) and may also justify the upregulation of this gene in all strains during PEC infection because the duration of infection was 48 h (Fig. 1E).

### *Mycobacterium tuberculosis* clinical strains show differential growth rates in the presence of cholesterol

The growth of the H37Rv wild-type strain corresponds with experimental work conducted by Pandey and Sassetti (2008) and Pawełczyk et al. (2021), with similar consumption of cholesterol noted. Results obtained from the growth curve showed the KZN1435 and F11 strains exhibited higher growth compared with the Beijing and KZN605 strains (Fig. 3A and B). These results were supported by a pan-genomic study, encompassing the analysis of genomic sequences, which identified 30 strain-specific genes belonging to KZN1435 and F11 clinical strains that were enriched in the lipid metabolism and transport category, implying that these strains are better ‘equipped’ for lipid metabolism compared with other clinical strains (Yang et al. 2018). Another study analysing genomic sequences, performed by Xu et al. (2013), indicated that the KZN1435 and F11 strains possessed 260 and 264 essential genes, respectively, with higher predicted lipid metabolic functions than the 257 genes present in the laboratory H37Rv strain. Granted that the clinical strains mimicked the growth patterns in cholesterol media when compared with 7H9, the strains still maintained high bacillary numbers in the presence of cholesterol, despite being a single carbon source assay. There also appears to be a correlation between highly virulent isolates of *M. tuberculosis* and the ability to grow faster (Theus et al. 2005). Additionally, a higher bacterial burden has been exhibited to cause increased lung damage and higher mortality (Manca et al. 2001, Dormans et al. 2004). The F11 strain has been found to be widely distributed in the Western Cape communities of South Africa (Warren et al. 1999, 2002). While it has a high occurrence in this province, it must be cautioned that it does not necessarily translate to higher virulence, and this remains to be determined. The elevated sequestering of cholesterol by these strains is especially important considering that *M. tuberculosis* has access to a reservoir of cholesterol, originating from the host and then feeding into mycobacterial central carbon metabolism (Pandey and Sassetti 2008, Yang et al. 2009), with *M. tuberculosis* itself inducing a foamy macrophage phenotype that is loaded with accumulated host lipid droplets (Peyron et al. 2008).

KZN605 and Beijing strains were shown to be slow growers in cultures supplemented with cholesterol. Utilisation of cholesterol at a slower rate could infer that these strains are more prone to survive longer in the host in a persistent state, leading to increased pathogenicity. Cholesterol utilisation in *M. tuberculosis* has been linked to both persistence and dormancy (Pandey and Sassetti 2008, Soto-Ramirez et al. 2017). Modern Beijing strains have been reported to accumulate a high amount of triacyglycerols (TAG), a macrolipid by-product of cholesterol metabolism and functioning as an energy reserve during dormancy (Daniel et al. 2004, Deb et al. 2009, Tong et al. 2020). The synthesis of the TAG molecule conveys to the Beijing strain a bonus competitive advantage in addition to degrading cholesterol at a slower rate, to be able to endure under persistent/latent conditions.

### RNA-seq analysis and pathway mapping reveal certain lipid-metabolic pathways to be differentially expressed in clinical strains of *M. tuberculosis*

RNA-seq analysis has revealed the transcriptomic signatures of genes involved in various lipid-metabolic pathways in the different clinical strains of *M. tuberculosis* (Fig. 4A). The process of cholesterol degradation involves two main major pathways, that is, the breakdown of the aliphatic side and the disintegration of the A, B, C and D rings. These processes are said to occur simultaneously of each other (Ouellet et al. 2011). A single enzyme, KSH, is encoded for by a two-component system, an oxygenase (*kshA*) and a reductase (*kshB*), and is responsible for the degradation of the sterol ring (Van der Geize et al. 2007), with *kshB* being additionally responsible for the initial stages of β-oxidation of the side chain (Capyk et al. 2009). Numerous studies have proven the *kshA* and *kshB* genes to be indispensable for *M. tuberculosis* infection in murine and macrophage models (Dormans et al. 2004), leading to the conclusion that metabolism of cholesterol during infection without the functioning of these genes is unachievable (Griffin et al. 2011). Additionally, deletion mutants for *kshA*/*B* in *M. tuberculosis* H37Rv showed that the strain was incapable of metabolising cholesterol (Hu et al. 2010), extending the importance of these genes in the direct breakdown of cholesterol as a primary carbon source. The current study, which compared the expression of cholesterol degradative genes in the clinical strains (Beijing and KZN605) with the laboratory H37Rv strain, established that *kshA* was significantly upregulated in both clinical strains (Fig. 4A). *kshA* has been shown to be induced upon a nutrient starvation state (Betts et al. 2002), which is possibly why the Beijing and KZN605 strains showed an upregulation of this gene. This further reiterates the notion that the slow growers, Beijing and KZN605, were approaching or in a persistent state in high cholesterol conditions. Future studies will subject these clinical strains of *M. tuberculosis* in minimal media in the presence of rifampicin to reveal their ability to persist in a nutrient-deprived environment using cholesterol as the main carbon source to support the current hypothesis, as recently revealed by Lata et al. (2022). Furthermore, *kshA* has been evidenced to be upregulated in a modern Beijing strain in Thailand, which was termed a ‘superspreader’ (Aiewsakun et al. 2021). The present study showed a downregulation of *kshB* in Beijing (Fig. 3A). However, it is important to note that this expression was relative to the expression levels of H37Rv in the same nutrient conditions, thereby eliciting the conjecture that the wild-type strain had a higher expression of *kshB* than Beijing. This result was confirmed by qRT-PCR analysis (Fig. 5A), where *kshB* exhibited the highest expression levels in H37Rv (significantly) during growth in a cholesterol medium than both Beijing and KZN605. The result concurs with a previous study where *kshB* was shown to be upregulated in the wild-type H37Rv, in the same profiling condition (Pawełczyk et al. 2021).

The *fadD18* gene possesses the function of acyl-CoA ligase and releases a unit of acetyl-CoA (Kapopoulou et al. 2011). Initially, this gene was previously shown to be upregulated in H37Rv when cultivated on 1% cholesterol (Nesbitt et al. 2010), a higher concentration than what was used in this study (0.01%). In this experimental work, remarkably, *fadD18* was downregulated in the H37Rv wild-type strain in the cholesterol versus 7H9 condition but opposingly upregulated in Beijing (Fig. 4A). It is quite possible that because other paralogues of the 36 acyl-CoA ligase genes with the same function exist (Trivedi et al. 2004), this role was undertaken by these other proteins for H37Rv, particularly for this lower concentration of cholesterol. This necessitates further studies on elucidating the role of other genes within this group or determining other potential roles of fadD genes in lipid metabolism, as they have been shown to take on diverse roles in lipid-metabolic categories other than cholesterol degradation (Portevin et al. 2005, Lynett and Stokes 2007).

The first step of side-chain β-oxidation is constituted by the acyl CoA-dehydrogenases (Cole et al. 1998). The *fadE24* gene was upregulated for KZN605 when compared with H37Rv (Fig. 4A). Transcriptional evaluation of an isoniazid-treated (first-line anti-TB drug (Bernstein et al. 1952, Fox 1952)) *M. tuberculosis* strain revealed the increased expression of *fadE24*. The activation of this gene could conjecture the involvement of said gene in isoniazid-resistant mechanisms (Vilchèze and Jacobs Jr, 2014). The upregulation of the *fadE26* gene was noted in the infection of *M. tuberculosis* clinical strains in murine bone marrow-derived macrophages (Homolka et al. 2010), during pulmonary TB infection (Rachman et al. 2006) and in H37Rv grown in cholesterol (Pawełczyk et al. 2021). In the current study, the *fadE26* gene was found to be upregulated in the MDR Beijing strain and downregulated in the KZN605 strain, comparable with H37Rv (Fig. 4A). Consistent with these results, a modern MDR Beijing strain exhibited an upregulation of *fadE26* in relation to the H3Rv reference strain during infection of THP-1 macrophage cells (Aiewsakun et al. 2021).

The degradation of cholesterol permits the generation of three intermediates: propionyl-CoA, acetyl-CoA and pyruvate. The amount of these intermediates is based on the regulation of genes in the preceding steps. The intermediates are critical in fuelling various pathways, one such pathway being the mycolic acid synthesis pathway (Savvi et al. 2008). Propionyl-CoA is one such major metabolite, which activates the methylmalonyl pathway that in turn will activate the FAS I pathway (synthesis of fatty acids) or the FAS II pathway (synthesis of mycolic acids) (Portevin et al. 2004), both ultimately responsible for the production of the building blocks for mycolic acids (Marrakchi et al. 2014). The *accA3* gene encodes for an acetyl‐CoA carboxylase, and in conjunction with *accD6*, oversees the conversion of acetyl-CoA (another intermediate metabolite) to malonyl-CoA (Ehebauer et al. 2015). In this study, *accA3* was shown to be significantly upregulated in the H37Rv control strain (H37Rv cultured in typical culturing medium being the comparative standard) (Fig. 4B). Increased production of malonyl-CoA serves as an elongation unit and promotes fatty acid biosynthesis (Oh et al. 2006). The simultaneous upregulation of the *accD5* gene in H37Rv is noteworthy (Fig. 4B), as *accA3* and *accD5* are proficient at forming a complex that can act on propionyl-CoA as well, and in turn synthesise methylmalonyl-CoA. This intermediate is then utilised to produce integral mycobacterial cell wall lipids. Expression data for this gene, as generated by qRT-PCR (Fig. 5B), confirms the increased expression of H37Rv when evaluated against the same strain in 7H9 medium, while additionally showing high expression of Beijing and a lower expression of KZN605. Malonyl-CoA can either enter the FAS I or FAS II pathways (Lin et al. 2006, Oh et al. 2006, Ehebauer et al. 2015). The *fas* gene, which encodes for a fatty acid synthase, is implicated in the FAS I pathway and was shown to be upregulated for the Beijing and H37Rv strains and downregulated for the KZN605 strain (Fig. 4B). The *fas* gene or *Rv2524c* encodes for a polypeptide that is fundamental for the functioning of the FAS I system, which generates fatty acids that are thereafter directed into the FAS II cycle (Smith et al. 2003). The high fold change observed for Beijing for this gene echoes the reputation of the Lineage 2 Beijing strain that is associated with drug resistance (Hanekom et al. 2011), as mycolic acid patterns have a direct effect on the emergence of this resistance (Müller et al. 2013). A first line anti-TB drug, pyrazinamide, explicitly targets the operation of the FAS I system (Zimhony et al. 2000). For drug-resistant strains such as Beijing, this proves to be a promising drug target. Interestingly, the downregulation of the *accD4* gene was detected in Beijing for the conversion of meromycolates to α-Mycolate. This directly conflicts with the upregulated *fas* gene in the FAS I system. It is hypothesised that the decreased expression of this gene might be due to a mutation within the gene or in other genes that are part of the same operon. One such study showed that introducing a mutation to the *pks13* gene in *Corynebacterium glutamicum*, which shares a high sequence identity with mycobacteria for FAS II associated genes, directly affected the expression of *accD4* (Portevin et al. 2004). The *pks13* gene is neighbour to *accD4* and works mutually in yielding mature mycolic acids (Portevin et al. 2004, 2005). Malonyl-CoA entering the FAS II pathway involves the elongation of meromycolate intermediates, which are catalysed by *kasA* and *kasB* (Kremer et al. 2002, Slayden and Barry 3rd 2002). The *kasB* gene showed an upregulation in the H37Rv strain (Fig. 3B), an indicator of longer mycolic acid chain length. Regulating the expression of these genes would be beneficial in controlling the adverse effects of infection, as evidenced when a *kasB* mutant strain persisted in mice, neither causing disease nor death (Bhatt et al. 2007).

TAG synthesis is the breakdown of free fatty acids, which are stored as triacylglycerol. This storage is an indicator of a dormancy model (Daniel et al. 2004). The downregulation of the *tgs1* gene, an important contributor to TAG synthesis, demonstrates that the H37Rv strain was not reaching or in a dormant state (Fig. 4C). However, the significant upregulation of a lipase, *lipL*, in KZN605 (Fig. 4C), expresses the need for energy and acetyl-CoA accumulation, products of TAG degradation (Shi et al. 2010). Lipases are considered critical virulence factors during *M. tuberculosis* infection (Rodríguez et al. 2014, Vromman and Subtil 2014). These results concur with the previous statements, that it is conceivable that H37Rv actively replicated in cholesterol (thereby not requiring the need to induce dormancy-related genes) and KZN605 in an extremely slow growth state, was less adept or exceedingly slow at sequestering cholesterol, thereby expressing the need for hydrolysis of TAG for nutrients. The Beijing strain, being a faster grower in cholesterol than KZN605, exhibited a downregulation for both lipases (Fig. 4C). Hypervirulent strains affiliated with the W-Beijing lineage have been reported to accumulate TAG during active growth (Reed et al. 2007), leading to the conclusion that Beijing might store TAG, but does not require its hydrolysis, possibly until complete depletion of cholesterol.

The synthesis of sulfolipid-1 (SL-1) branches from the methylmalonyl pathway, a consequence of propionyl-CoA being incorporated into cell wall virulence lipids (Rainwater and Kolattukudy 1983). The *pks2* gene, a polyketide synthase, was proven to be essential for SL-1 synthesis (Sirakova et al. 2001). The *mmpl8* gene, implicated in the transportation and a precursor step for SL-1 synthesis, is similarly needed (Converse et al. 2003). The *pks2* gene has been highlighted as being necessary for growth on cholesterol (Griffin et al. 2011), while *mmpL8* is regarded as being vital for *M. tuberculosis* to thrive during innate immune responses and establishment of infection of mice at a high level (Converse et al. 2003). The promotion of SL-1 synthesis in KZN605 and H37Rv is of striking importance (as shown by the increased expression of both *pks2* and *mmpL8* in these strains) (Figs 4D, 5C and D). For years, the connection between sulfolipids and *M. tuberculosis* virulence had eluded scientists, with certain studies indisputably showing sulfolipid synthesis having no bearing on *M. tuberculosis* virulence, reportedly in animal models of infection (Rousseau et al. 2003). However, a study showed that sulfolipid-deficient strains had higher intracellular survival in human and not mouse macrophages, establishing that sulfolipid synthesis as a virulence determinant is host-specific, and ultimately diminishes *M. tuberculosis* virulence (Gilmore et al. 2012). A Beijing strain has been described to encompass a high amount of phenolic glycolipid and triglycerides in its cell envelope, leading to the assumption that the decreased expression of *pks2* and *mmpL8* (Figs 4D, 5C and D) in Beijing could be interpreted as Beijing focusing its efforts on synthesising these cell wall components instead (Reed et al. 2004, 2007).

Profiling these genes in genetically diverse clinical strains of *M. tuberculosis*-infected PECs and in an *in vitro* lipid model offers more critical insights into the regulation of the lipid-associated virulence factors in *in vitro* models. Elucidating proposed phenotypes for these strains, because of such genetic modifications, may prove useful in the development of anti-TB therapeutics that is strain specific. A limitation to this study is the lack of an avirulent control, such as the *M. tuberculosis* H37Ra strain; however, virulent-specific traits in the transcriptome changes are implicated for lipid metabolism for KwaZulu-Natal (South Africa)-dominant strains of *M. tuberculosis* (Pillay and Sturm 2007, Chihota et al. 2012).

## Conclusions

One of the characteristic features of *M. tuberculosis* is its ability to utilise host-derived lipids as a source of carbon during infection. However, studies pertaining to the role of lipid metabolism in clinical isolates of *M. tuberculosis* have been inadequate. The current study profiled transcriptomic changes of *M. tuberculosis* grown on standard 7H9 broth, infecting PECs, and lipid-rich media, and the findings revealed that the diversity encountered in clinical strains may account for variation in regulation of lipid-metabolic transcripts in KZN605 and Beijing strains, suggesting a potential link to the virulence of these strains. Furthermore, changes in genes associated with cholesterol and fatty acid metabolism varied among the KZN605 and Beijing strains, suggesting strain-specific behaviour in lipid metabolism. This study provides lipid-specific transcriptome profiles in clinical strains of *M. tuberculosis* that can be implicated for virulence traits, survival, persistence and the replication of these strains in *in vitro* and *in vivo* infection models.

## Data Availability

Raw RNA sequencing data is available upon request from the corresponding authors.
